# Assessment of clinical outcomes in patients with inflammatory arthritis: analysis from the UK Medical Cannabis Registry

**DOI:** 10.1097/YIC.0000000000000556

**Published:** 2024-07-02

**Authors:** Ann Francis, Simon Erridge, Carl Holvey, Ross Coomber, Rahul Guru, Alia Darweish Medniuk, Mohammed Sajad, Robert Searle, Azfer Usmani, Sanjay Varma, James Rucker, Michael Platt, Wendy Holden, Mikael H. Sodergren

**Affiliations:** a Department of Surgery and Cancer, Medical Cannabis Research Group, Imperial College London; b Department of Medicine, Curaleaf Clinic; c Department of Trauma and Orthopaedics, St. George’s Hospital NHS Trust, London; d Department of Pain Management, Cardiff and Vale University Health Board, Cardiff; e Anaesthetic Department, Southmead Hospital, North Bristol NHS Trust, Bristol; f Department of Psychological Medicine, Kings College London; g Centre for Affective DIsorders, South London & Maudsley NHS Foundation Trust, London, UK

**Keywords:** arthritis, cannabidiol, chronic pain, medical cannabis, tetrahydrocannabinol

## Abstract

The aim of this study was to assess changes in validated patient-reported outcome measures after initiation of cannabis-based medicinal products (CBMPs) and the safety of CBMPs in patients with inflammatory arthritis. A prospective case series from the UK Medical Cannabis Registry was analyzed. The primary outcomes changes were in Brief Pain Inventory, McGill Pain Questionnaire, EuroQol 5-dimension 5-level (EQ-5D-5L), Generalised Anxiety Disorder-7 questionnaire, and Single-Item Sleep Quality Scale at 1, 3, 6, and 12 months of follow-up compared with baseline. Adverse events were analyzed in accordance with Common Terminology Criteria for Adverse Events, v.4.0. Statistical significance was defined as a *P*-value less than 0.050. Eighty-two patients met the inclusion criteria. Initiation of CBMP treatment was associated with improvements in Brief Pain Inventory, McGill Pain Questionnaire, EQ-5D-5L, Generalised Anxiety Disorder-7 questionnaire, and Single-Item Sleep Quality Scale at 1, 3, 6, and 12 months compared with baseline (*P* < 0.050). There were 102 (44.35%) mild adverse events, 97 (42.17%) moderate adverse events, and 31 (13.48%) severe adverse events recorded by 21 (25.61%) participants. This study suggests that CBMP treatment is associated with pain improvement and increased health-related quality of life for inflammatory arthritis patients. While causality cannot be inferred in this observational study, the results support the development of randomized control trials for inflammatory arthritis pain management with CBMPs.

## Introduction

Inflammatory arthritis (IA) is a group of diseases of which rheumatoid arthritis, psoriatic arthritis, and ankylosing spondylitis are the most common (Hoving *et al*., 2014). The prevalence of inflammatory arthritis is estimated to be around 3% with rheumatoid arthritis affecting 1% of the global population (Braun *et al*., 1998; Gabriel, 2001). The impact of inflammatory arthritis on individuals is significant, and increasing, with disability-adjusted life-years increasing by 12% between 1990 and 2010 (Murray *et al*., 2013). Chronic pain secondary to inflammatory arthritis can be disabling and is associated with poor sleep quality, fatigue, and reduced cognitive function (Fitzcharles and Shir, 2008; Pitcher *et al*., 2019). Comorbid mental health conditions in patients with inflammatory arthritis are therefore common, with the prevalence of depressive disorder in people with rheumatoid arthritis estimated at 13–20% (Sheehy *et al.*, 2006; Treharne *et al*., 2007). inflammatory arthritis also has a societal impact; 20–35% of patients have to stop working 2–3 years after disease onset (Jäntti *et al*., 1999; SOKKA *et al*., 1999; Barrett *et al*., 2000).

Inflammatory arthritis management is multidisciplinary and medications to treat inflammatory arthritis are split into disease-modifying antirheumatic drugs (DMARDs), non-steroidal anti-inflammatory drugs (NSAIDs), and corticosteroids (Wood and O’Dell, 2004). NSAIDs and cyclooxygenase-2 (COX-2) inhibitors are most commonly used for alleviating pain related to inflammatory arthritis. NSAIDs, however, are associated with an increased risk of gastrointestinal ulcers, perforation, and hemorrhage (Bobek *et al.*, 2022); every year around 1.5% of patients with rheumatoid arthritis are hospitalized with gastrointestinal problems (Singh, 1998). While COX-2 inhibitors reduce the risk of gastrointestinal ulcers, they have an increased risk of thrombotic events compared with NSAIDs (Strand and Hochberg, 2002). Both COX-2 inhibitors and NSAIDs have been associated with an increased risk of fluid retention and impairment of renal function in susceptible patients (FitzGerald and Patrono, 2001). Finally, many patients with inflammatory arthritis-associated chronic pain are prescribed opioids for pain relief, however, opioids have a high side-effect profile, with dependency being a particular concern (Benyamin *et al*., 2008). Hence, there is a need for better therapeutic options for chronic inflammatory pain as many are not effective, have a significant side-effect profile, or are not appropriate for long-term use (McCracken, 2023).

Cannabis-based medicinal products (CBMPs) derived from the cannabis plant have been identified as novel therapeutics for inflammatory arthritis-associated chronic pain due to their ability to modulate the endocannabinoid system (ECS) (Khoury *et al.*, 2022). The ECS consists of cannabinoid receptors, endocannabinoids (endogenous ligands of cannabinoid receptors), and enzymes (Di Marzo, 2008). Cannabinoid receptor 1 (CB1) and cannabinoid receptor 2 (CB2) are G protein-coupled receptors, which are expressed on chondrocytes and osteocytes (La Porta *et al*., 2015). CB1 and CB2 receptors have been implicated in the maintenance of joint homeostasis in both healthy joints and those affected by inflammatory arthritis (Gui *et al*., 2014; Sido *et al.*, 2015; Dunn *et al*., 2016). There is evidence suggesting CB1 facilitates the adhesions of fibroblast-like synoviocytes to fibronectin, reducing migratory capacity and potentially reducing cartilage destruction (Sido *et al.*, 2015; Dunn *et al*., 2016). There are also increased CB2 levels in fibroblast-like synoviocytes in patients with rheumatoid arthritis compared with osteoarthritis, indicating CB2 involvement in inflammatory arthritis pathophysiology (Gui *et al*., 2014).

Cannabinoid receptors, in particular CB2, have also been demonstrated to have immunoregulatory effects (Barrie and Manolios, 2017). The most abundant active phytocannabinoids found in CBMPs are (−)-trans-Δ9-tetrahydrocannabinol (THC) and cannabidiol (CBD) (Pertwee, 2007). THC is predominantly a partial CB1 receptor agonist, while CBD acts to increase the available concentrations of endogenous cannabinoids (Pertwee *et al*., 2010), by inhibiting their breakdown (Leweke *et al.*, 2012; Elmes *et al*., 2015). These effects, in addition to off-site actions at serotonin and transient receptor potential channels, have been implicated in reducing the transmission of nociceptive signals, as well as modifying the emotional and cognitive aspects of chronic pain (Maldonado *et al.*, 2016). Despite this, there is a dearth of clinical evidence on the effects of CBMPs on disease modification (Barrie and Manolios, 2017). While the evidence does demonstrate an effect of noninhaled CBMPs on chronic pain, the evidence is subject to significant heterogeneity (Wang *et al*., 2021). There are no randomized controlled trials greater than 4 weeks in duration detailing outcomes on inhaled CBMPs, and only one study of 58 patients examining the effects of a CBMP in rheumatoid arthritis (Blake *et al*., 2006; Wang *et al*., 2021).

This study primarily aimed to assess changes in pain-specific and general health-related quality of life (HRQoL) measures in inflammatory arthritis patients from the UK and prescribed a range of CBMPs. The secondary aim was to assess the incidence of adverse events to characterize the safety profile of CBMPs in inflammatory arthritis patients.

## Methods

### Study design

This formal, sequential clinical case series investigated the effects of prescribed CBMPs in inflammatory arthritis patients utilizing data from the UK Medical Cannabis Registry (UKMCR). This observational study followed STROBE guidelines (von Elm *et al*., 2008). Ethical approval was granted by the Central Bristol Ethics Committee (22/SW/0145). All participants were enrolled consecutively and provided written informed consent. Data were collected remotely whereby patients completed PROMs and adverse event questionnaires electronically via an online web-based platform at 1, 3, 6, and 12 months.

### Settings and participants

The UKMCR enrolled its first patients in December 2019 and collects longitudinal pseudonymized data from patients in the UK and Channel Islands prescribed CBMPs.

Individuals aged at least 18 years with a primary diagnosis of inflammatory arthritis-associated chronic pain met the inclusion criteria. Exclusion criteria included patients who had not completed a baseline PROM assessment and less than 12 months of enrolment in the Registry. Data was extracted on 9 January 2023. CBMPs adhered to Good Manufacturing Practice standards and were prescribed by a specialist after approval by a multidisciplinary committee (Medicines and Healthcare Products Regulatory Agency, 2020).

### Data collection

The following baseline demographic data were collected: age, sex, occupation, and body mass index (BMI) (kg/m^2^). Other indications for treatment with CBMPs and comorbidities were also recorded. The Charlson Comorbidity Index, a prognostic tool commonly used in observational studies, was calculated for each participant (Brusselaers and Lagergren, 2017).

Tobacco, alcohol, and cannabis status at baseline were collected, including smoking status, pack-years, weekly alcohol consumption (units), cannabis use status, frequency of cannabis use, and current quantity of cannabis intake (grams). To quantify the individual history of illicit cannabis use, a metric of ‘cannabis gram years’ was used (Erridge *et al*., 2021). The following CBMP prescription details were collected at baseline and follow-up intervals: company, formulation, route of administration, CBD dose/day (mg), and THC dose/day (mg). Participants were strongly counseled against continuing to consume illicit cannabis by the treating physician.

Patient medication data, including drug names, medicine doses per 24 h, and prescription start/end dates was recorded. Medication names were mapped to SNOWMED CT codes to maintain uniformity (Lee *et al*., 2014). The British National Formulary conversion factors were used to calculate oral morphine equivalents (OMEs) for opioid medications (British National Formulary, 2023).

#### Patient-reported outcome measures

All patients had the following PROMs recorded at baseline and all follow-up intervals: Brief Pain Inventory (BPI), Short-Form McGill Pain Questionnaire (SF-MPQ-2), General Anxiety Disorder-7 (GAD-7), Single-item Sleep Quality Scale (SQS), and the EuroQol 5-dimension 5-level (EQ-5D-5L).

*Pain-specific patient-reported outcome measures:* The BPI is a two-part PROM that assesses pain severity and interference using 11 categories (Kapstad *et al.*, 2010; Jumbo *et al*., 2021). Pain severity and interference are ranked on a scale of 0–10. Pain severity ranges from ‘0’ = ‘no pain’/‘no interference’ to ‘10’ = ‘pain as awful as you can imagine’/‘complete interference’ (Jumbo *et al*., 2021). A minimal clinically important difference in BPI pain severity is defined as a one-point improvement (Dworkin *et al*., 2008).

SF-MPQ-2 assesses pain across 22 questions according to four major subscales continuous, intermittent, neuropathic, and affective (Dworkin *et al*., 2009). Each subscale is rated on a scale of 0–10: whereby 0 = “no pain” and 10 = “worst pain”. Each subscale score is the mean of its specific descriptors, whereas the overall SF-MPQ-2 score is the mean score of the subscales (Dworkin *et al*., 2009; Hawker *et al*., 2011).

*Health-related quality of life–specific patient-reported outcome measures:* GAD-7 is a PROM designed to screen and measure the severity of generalized anxiety disorder (Spitzer *et al*., 2006). Participants report how frequently they were affected by core generalized anxiety disorder symptoms over the past 2 weeks. The scale ranges from 0 to 21, with scores of ≥5, ≥10, and ≥15 signifying mild, moderate, and severe anxiety symptoms, respectively (Spitzer *et al*., 2006; Löwe *et al*., 2008; Plummer *et al*., 2016).

The SQS assesses sleep quality. Participants rated overall sleep quality over the past 7 days as terrible (0), poor (1–3), fair (4–6), good (7–9), or excellent (10) (Yi *et al*., 2009; Snyder *et al*., 2018).

The EQ-5D-5L evaluates general HRQoL across mobility, self-care, usual activities, pain/discomfort, and “anxiety/depression domains utilizing a 1 (no problems) to 5 (extreme problems) scale (van Hout *et al*., 2012). The resulting health state is mapped to EQ-5D-5L index values validated for a UK population (van Hout *et al*., 2012; National Institute for Health and Care Excellence, 2019). Optimum health is assigned an index score of 1, while an index score <0 represents a health state worse than death (van Hout *et al*., 2012).

#### Adverse events

adverse events were recorded throughout treatment with CBMPs through contemporaneous self-reporting, or through direct questioning during completion of PROMs or during a clinical consultation. They were reported according to the Common Terminology Criteria for Adverse Events, version 4.0 [Common Terminology Criteria for Adverse Events (CTCAE) Version 4.0, 2009].

### Statistical methods

Clinicopathological drug and alcohol data were assessed using descriptive statistics.

Demographic data are presented as the mean ± SD, median [interquartile range (IQR)], or frequency (%), as appropriate.

Longitudinal changes in PROMs were analyzed using repeated-measures analysis of variance with pairwise analysis of statistically significant values conducted using post-hoc analysis with Bonferroni correction. If PROM data was missing during the follow-up period, it was handled using the baseline observation carried forward approach (Liu-Seifert *et al*., 2010). Changes in opiate prescribing were analyzed utilizing a paired *t*-test analysis of OMEs at baseline and 12 months.

All statistical analysis was conducted using Statistical Package for Social Sciences (SPSS) (version 29.0.0.0; IBM Statistics, Armonk, New York, USA). Statistical significance was defined as a *P*-value less than 0.050. Graphs were produced with GraphPad Prism [version 9.5.1 (528) for macOS; GraphPad Software Inc., San Diego, California, USA].

## Results

### Patient data

At the time of data extraction, 9464 patients were registered in the UKMCR. In all, 9382 (99.13%) patients were excluded: those who were treated for less than 12 months (*n* = 6404; 67.67%), those without baseline PROMs (*n* = 980; 10.36%), and without a diagnosis of inflammatory arthritis (*n* = 1996; 21.09%). Hence, 82 patients were ultimately included in this study.

Baseline demographic details of all patients included in the analysis are presented in Table 1. The mean age of patients was 47.61 ± 14.31 years and the male-to-female ratio was 1 : 1. The mean BMI was 30.06 (±6.70) kg/m^2^ and the most frequent occupation reported was “Unemployed” (*n* = 41, 50.00%).

**Table 1:** Demographic details of patients at baseline assessment

Demographic details	*n* (%)/mean (±SD)
Sex	
Male	41 (50.00)
Female	41 (50.00)
Age (years)	47.61 ± 14.31
BMI (kg/m^2^)	29.95 ± 7.15
Occupation	
Clerical support workers	2 (2.44)
Craft and related trades workers	2 (2.44)
Elementary occupations	3 (3.66)
Managers	6 (7.32)
Plant and machine operators, and assemblers	2 (2.44)
Professional	15 (18.29)
Service and sales workers	2 (2.44)
Technicians and associate professionals	1 (1.22)
Other occupations	6 (7.32)
Unemployed	41 (50.00)

The baseline tobacco, alcohol, and cannabis status of patients are presented in Table 2. Baseline analysis revealed that many patients were current cannabis consumers (*n* = 39, 47.56%) at baseline, with the majority consuming cannabis daily (*n* = 37, 94.88%). The median daily quantity of cannabis consumed was 1.25 (IQR: 0.75–2.00) g/day. The median lifetime cannabis consumption of patients who were current cannabis users was 10.00 (IQR: 3.00–26.00) gram years. The remaining patients were either ex-users (*n* = 12, 14.63%) or cannabis-naive (*n* = 31, 37.80%).

**Table 2:** Tobacco, alcohol, and cannabis status of study participants

Tobacco, alcohol, and cannabis status		*n* %/median (IQR)
Tobacco status	Current smoker	14 (17.07)
	Pack years	7.5 (3.00–30.00)
	Ex-smoker	41 (50.00)
	Pack years	10.00 (4.00–20.00)
	Nonsmoker	27 (32.93)
Weekly alcohol consumption (Units)		0.00 (0.00–2.25)
Cannabis status	Current user	39 (47.56)
	Current quantity of cannabis consumption (g/day)	1.25 (0.75–2.00)
	Lifetime quantity of cannabis consumption (gram years)	10.00 (3.00–26.00)
	Ex-user	12 (14.63)
	Lifetime quantity of cannabis consumption (gram years)	1.50 (1.00–5.25)
	Nonuser	31 (37.80)
Frequency of cannabis use for current users	Every day	37 (94.88)
	Every other day	1 (2.56)
	1–2 times per week	1 (2.56)
	<1 times per month	0 (0)

IQR, interquartile range.

### Cannabis-based medicinal products

CBMP dosing is displayed in Table 3. Most patients (*n* = 79; 94.81%) were prescribed both CBD and THC. Of the remaining patients, three (1.89%) were prescribed THC only. The median dose of CBD and THC was 20.00 (20.00–35.00) mg/day and 110.00 (10.00–222.90) mg/day, respectively. The most commonly prescribed treatments were Adven 20 and 50 sublingual oils and Adven EMT1 flos (Curaleaf International, Guernsey, UK).

**Table 3:** Details of cannabis-based medicinal product prescribed for study participants (*N* = 159)

CBMP dosing	*n*/*N* (%)/median (IQR)
Cannabinoid contents	
Number of patients prescribed CBD alone	0 (0.00)
Number of patients prescribed THC alone	3 (3.66)
Number of patients prescribed both CBD and THC	79 (94.81)
Administration route	
Number of patients using sublingual/oral formulations only	28 (34.15)
Number of patients using vaporized flower only	17 (20.73)
Number of patients using both sublingual/oral formulations and vaporized flower	37 (45.12)
Dosage	
CBD dosage (mg/day)	20.00 (20.00–35.00)
THC dosage (mg/day)	111.00 (10.00–222.90)

CBD, cannabidiol; CBMP, cannabis-based medicinal product; IQR, interquartile range; THC, (−)-trans-Δ9-tetrahydrocannabinol.

### Patient-reported outcome measures

**Fig. 1:**
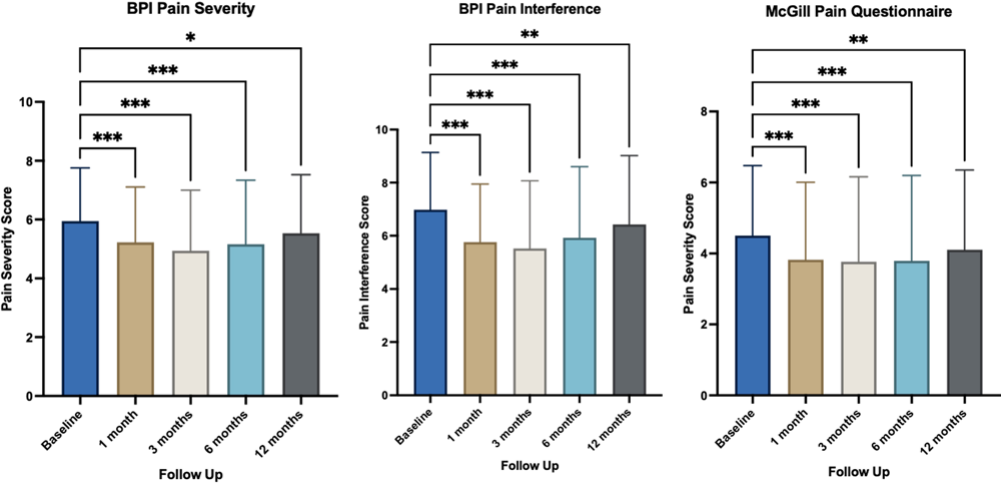
Paired baseline and follow-up scores for BPI and McGill Pain Questionnaire for inflammatory arthritis patients after 1, 3, 6, and 12 months of follow-up. Scores are presented as mean ± SD. BPI, Brief Pain Inventory Index, *P < 0.05; **P < 0.01; and ***P < 0.001.

Figure 1 outlines the paired results comparing the pain-specific PROMs at baseline to 1, 3, 6, and 12 months for inflammatory arthritis patients. Improvements were observed in BPI pain severity and interference scores as well as the SF-MPQ-2 inflammatory arthritis subgroups (*P* < 0.050) (Supplementary Appendix A, Supplemental digital content 1, http://links.lww.com/ICP/A137).

Table 4 displays HRQoL PROMs at baseline, 1, 3, 6, and 12 months. Improvements were observed in GAD-7, SQS, and the EQ-5D-5L index value. There was a statistically significant improvement in GAD-7, SQS, and EQ-5D-5L Index between each follow-up period and baseline (*P* < 0.050).

**Table 4:** Paired baseline and follow-up scores for health-related quality of life patient-reported outcome measures for inflammatory arthritis patients after 1, 3, 6, and 12 months of follow-up

Patient-reported outcome measures		Follow-up				
		Baseline	1 month	3 months	6 months	12 months
GAD-7	Score	6.72 ± 0.66	4.81 ± 0.51	4.92 ± 0.58	5.37 ± 0.61	6.04 ± 0.68
	*P*-value		0.002	0.019	0.021	0.043
SQS	Score	3.82 ± 0.25	5.50 ± 0.24	5.45 ± 0.26	5.15 ± 0.28	4.71 ± 0.27
	*P*-value		<0.001	<0.001	<0.001	0.009
EQ-5D-5L mobility	Score	3.05 ± 0.11	2.87 ± 0.09	2.88 ± 0.11	2.88 ± 0.12	3.10 ± 0.11
	*P*-value					
EQ-5D-5L self-care	Score	2.39 ± 0.11	2.34 ± 0.11	2.18 ± 0.11	2.23 ± 0.11	2.39 ± 0.11
	*P*-value					
EQ-5D-5L usual activities	Score	3.22 ± 0.11	2.89 ± 0.10	2.79 ± 0.11	2.87 ± 0.12	3.07 ± 0.11
	*P*-value		0.007	<0.001	<0.001	0.702
EQ-5D-5L pain and discomfort	Score	3.68 ± 0.10	3.16 ± 0.09	3.05 ± 0.11	3.21 ± 0.11	3.44 ± 0.11
	*P*-value		<0.001	<0.001	<0.001	0.021
EQ-5D-5L anxiety and depression	Score	2.46 ± 0.12	2.04 ± 0.10	2.13 ± 0.11	2.16 ± 0.11	2.32 ± 0.12
	*P*-value		<0.001	0.029	0.004	0.448
EQ-5D-5L index value	Score	0.31 ± 0.03	0.46 ± 0.03	0.46 ± 0.03	0.42 ± 0.03	0.36 ± 0.03
	*P*-value		<0.001	<0.001	<0.001	0.006

Scores are presented as mean ± SD.

Paired Bonferroni-corrected *P* values are only calculated for statistically significant values (*P* < 0.050) on repeated-measures analysis of variance.

EQ-5D-5L, EuroQol 5-dimension 5-level; GAD-7, General Anxiety Disorder-7, SQS, Single-Item Sleep Quality Scale.

### Oral morphine equivalent analysis

Forty (48.78%) patients were regularly prescribed opioid medicines. There was no significant reduction in OME doses between baseline and end of follow-up (12 months) after the commencement of CBMP treatment (198.72 ± 112.52 vs. 155.37 ± 79.95 *P* < 0.234).

### Adverse events

**Fig. 2:**
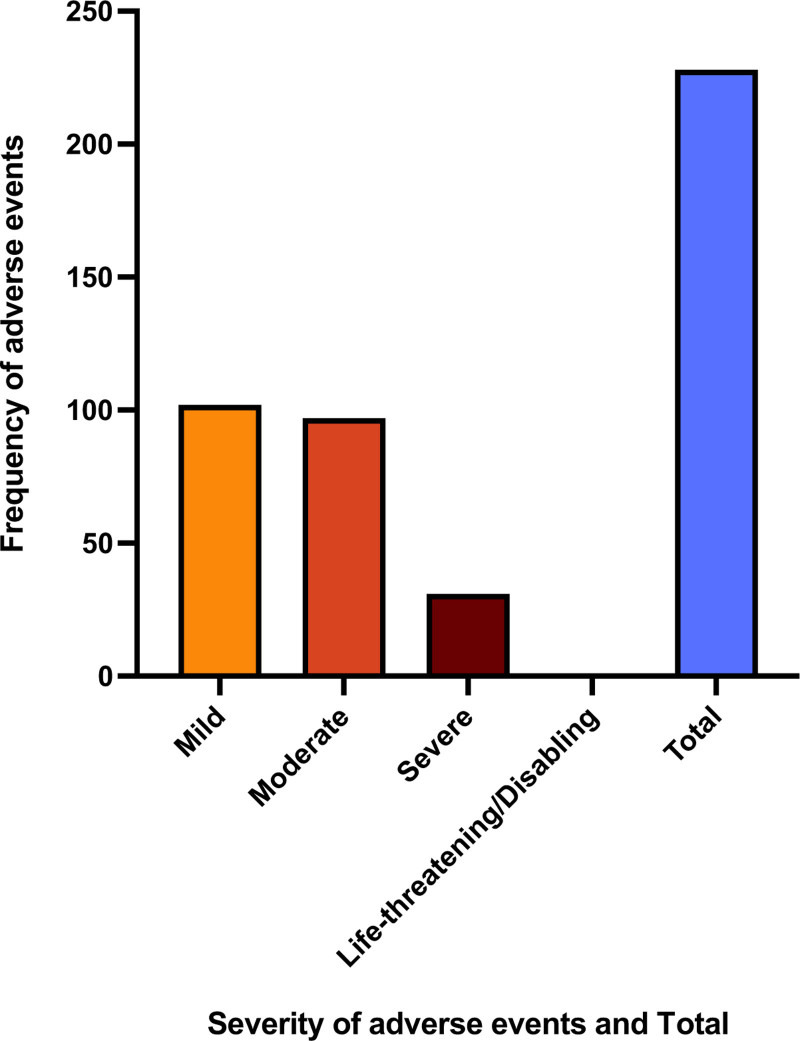
Adverse event frequency graded by severity for study participants from baseline to 12 months. The total number of adverse events (230; 280.49%) is also displayed.

Figure 2 displays the incidence of adverse events reported. A total of 230 (280.49%) adverse events were recorded by 21 (25.61%) patients. The most common adverse event was dry mouth (6.96%). There were 102 (44.35%) mild adverse events, 97 (42.17%) moderate adverse events, and 31 (13.48%) severe adverse events. There were no (0%) life-threatening adverse events reported by any of the study participants. For specific adverse events reported, see Supplementary Appendix B, Supplemental digital content 1, http://links.lww.com/ICP/A137.

## Discussion

This UKMCR prospective observational study of patients with inflammatory arthritis-associated chronic pain demonstrated improvements in all pain-specific PROMs in patients at 1, 3, 6, and 12 months of follow-up.

CBMP treatment was associated with reductions in pain-specific PROMs at all time points. Similar reductions in pain were observed in an observational study by Cahill *et al*. (2021) where improvements were reported in pain severity. The study length, however, was only 6 weeks with no long-term follow-up. This was corroborated by a randomized controlled trial by Blake *et al*. (2006) where patients with rheumatoid arthritis administered nabiximols experienced a reduction in pain severity as assessed using the SF-MPQ2 after 5 weeks of treatment. A meta-analysis conducted by Wang *et al*. (2021) found a 10% risk difference between individuals prescribed noninhaled CBMPs experiencing a clinically significant improvement in pain severity. This meta-analysis, however, was unable to include individuals prescribed dried flower CBMPs as no studies were identified with follow-up of at least 4 weeks (Wang *et al*., 2021). The present findings add further weight to the need to evaluate both oil-based and dried flower formulations of CBMPs to determine the optimum formulation in the setting of inflammatory arthritis-associated chronic pain.

CBMP treatment was also associated with improvement in generalized anxiety and sleep quality across all months of follow-up. Previous studies conducted using the UKMCR incorporating individuals with a broad range of indications for therapy with CBMPs have similarly reported improvements in sleep quality and generalized anxiety (Bapir *et al*., 2023; Olsson *et al*., 2023; Rifkin-Zybutz *et al*., 2023; Tait *et al*., 2023; Wan *et al*., 2023). The ECS has been heavily implicated in fear processing and sleep-promoting neural pathways (Lutz *et al*., 2015; Low *et al*., 2023). The clinical data is largely supportive of these effects, however, there is still a lack of consensus on the optimal preparations and dosing for individuals considering there appear to be bidirectional effects (Narayan *et al*., 2022). The mean baseline SQS and GAD-7 values in the present study suggest a proportion of individuals with inflammatory arthritis-associated chronic pain are affected by clinically significant anxiety and poor sleep quality, which should be considered within their pain management.

Across the 12-month follow-up period, improvements were observed in the EQ-5D-5L index for inflammatory arthritis patients (*P* = 0.006), implying an overall increase in patients’ HRQoL. A prior systematic review that aimed to assess the associated changes in HRQoL in individuals prescribed CBMPs found inconclusive evidence of an effect across all conditions (Goldenberg *et al*., 2017), In individuals, however, with chronic pain, there was a small positive impact on HRQoL (Goldenberg *et al*., 2017). This improvement is likely attributable to reductions in pain and discomfort as demonstrated by the improvement in the present study and those included in the review (Goldenberg *et al*., 2017). The EQ-5D-5L mobility and self-care scores, however, had no significant improvements over the follow-up period, as also shown in prior studies (Goldenberg *et al*., 2017). While improvement in reported pain severity is an important primary outcome, the lack of impact on mobility or self-care may suggest that the magnitude of this effect on HRQoL may be limited. Conversely, this may represent long-standing disability secondary to arthritic joint changes. Evaluation of HRQoL in randomized controlled trials may therefore benefit from inflammatory arthritis-specific and objective measures of disease activity in addition to pain severity.

### Limitations

The study is a limited case series which prevents establishing causality and reduces generalisability. It therefore remains uncertain whether improvements in pain and HRQoL were solely due to the CBMP treatment effect and not confounding factors. The study was susceptible to recall bias and subjectivity of PROMs may lead to differences in interpretation between participants. Moreover, the recall of associated PROMs may be influenced by the associated vasoactive and psychoactive effects of CBMPs, and positive media attention, which have been linked to an enhanced placebo effect and expectancy bias respectively (Vase *et al.*, 2014; Gedin *et al*., 2022). The expectancy bias could be enhanced further by the significant proportion of individuals who previously consumed cannabis, as well as the fact that CBMPs were accessed at a cost to the individual.

This study was subject to significant selection bias as treatment was limited to self-funded patients attending the same UK-based clinic. Paying for medical therapy also presents additional limitations as it has been shown to influence perceived efficacy (Díaz-Lago *et al.*, 2023). Moreover, almost half of all participants were current cannabis users (49.06%) at baseline, which could also affect perceptions of both the positive and negative effects of CBMPs. This may also affect opioid prescriptions, with patients titrating their opioid dose in response to self-treatment with illicit cannabis before receiving treatment with CBMPs. This may be a reason why there is discordance between the improvement in BPI pain severity and no significant reduction in OME dose per 24 h.

### Future scope

In a world of evolving precision medicine, there is potential for the involvement of CBMPs especially for inflammatory arthritis. CBMPs can be directly involved in disease-modifying therapy due to the implication of the ECS in the pathophysiology of inflammatory arthritis (Barrie and Manolios, 2017; Khoury *et al.*, 2022). At present, there is a paucity of information about which patient and treatment-specific factors influence the likelihood of a clinical response in inflammatory arthritis. This study would not be appropriately powered, however, future studies from the UKMCR should aim to incorporate a multivariable logistic regression analysis to understand the relationship between these factors and treatment success. This should include the effect of prior cannabis consumption on this cohort.

## Conclusion

This study demonstrates an associated improvement in pain severity and other relevant outcomes in individuals prescribed CBMPs for inflammatory arthritis-associated chronic pain. In addition, CBMPs were largely well tolerated by the majority of patients. While these results must be interpreted within the limitations of the study design, considering limited randomized controlled trial evidence on inflammatory arthritis-associated pain, these results provide further support for continued evaluation of CBMPs in this setting.

## Acknowledgements

Data that support the findings of this study are available from the UK Medical Cannabis Registry. Restrictions apply to the availability of these data. Data specifications and applications are available from the corresponding author.

All work was conducted at Curaleaf Clinic, London, UK.

All authors have contributed to and approved the final manuscript.

Ethical approval provided by South West–Central Bristol Research Ethics Committee (Reference: 22/SW/0145).

All participants completed written, informed consent before enrolment in the registry.

### Conflicts of interest

S.E., C.H., R.C., R.G., A.M., M.S., R.S., A.U., S.V., J.R., M.P., W.H., and M.H.S. are either employed by or provide clinical services on a consultancy basis to Curaleaf Clinic, London, UK. M.H.S. is a Chief Medical Officer of Curaleaf International. For A.F., there are no conflicts of interest.

## Supplementary Material


