# Supplementary material for: Assessment of clinical outcomes in patients with inflammatory arthritis: analysis from the UK Medical Cannabis Registry
Source: Int Clin Psychopharmacol. 2024 Jul 2;40(4):242–9. doi: 10.1097/YIC.0000000000000556 (PMC12122089; doi:10.1097/YIC.0000000000000556)
Supplement: Supplementary file 1 [file icp-40-242-s001.pdf]

Appendix

Appendix A: *Paired baseline and follow-up scores for pain-related Patient Reported Outcome Measures for inflammatory arthritis patients after 1,3,6 and 12 months of follow up. Paired Bonferroni-corrected P-values only calculated for statistically significant values (p<0.050) on repeated measures ANOVA. Scores presented as mean ± standard deviation.*
BPI – Brief Pain Inventory

Patient Reported Outcome Measures		Follow Up				
		**Baseline**	**1 month**	**3 months**	**6 months**	**12 months**
**BPI Pain Severity**	Score	5.95 ± 0.20	5.23 ± 0.21	4.94 ± 0.23	5.17 ± 0.24	5.53 ± 0.22
	p-value	-	0.001	<0.001	<0.001	<0.001
**BPI Pain Interference**	Score	6.98 ± 0.24	5.76 ± 0.24	5.53 ± 0.28	5.92 ± 0.30	6.43 ± 0.29
	p-value	-	<0.001	<0.001	<0.001	0.022
**McGill**	Score	4.50 ± 0.22	3.82 ± 0.24	3.77 ± 0.26	3.79 ± 0.27	4.11 ± 0.25
	p-value	-	<0.001	<0.001	<0.001	0.008

Appendix B: *Frequency of adverse events (AEs), separated by severity. AEs were recorded throughout the study, from baseline to 12 months. n = 448, mild – n = 178, moderate – n = 201, severe – n = 69*

	Severity of Adverse Event				
**Adverse Events**	**Mild**	**Moderate**	**Severe**	**Life-threatening/Disabling**	**Total (%)**
**Abdominal Pain**	5	1	0	0	**6 (2.60%)**
**Agitation**	0	0	0	0	**0 (0%)**
**Amnesia**	4	0	0	0	**4 (1.73%)**
**Anorexia**	2	4	1	0	**7 (3.04%)**
**Anxiety**	0	1	3	0	**4 (1.73%)**
**Ataxia**	3	1	0	0	**4 (1.73%)**
**Bloating**	0	1	0	0	**1 (0.43%)**
**Blurred Vision**	4	3	1	0	**8 (3.48%)**
**Chest Pain Cardiac**	0	1	0	0	**1 (0.43%)**
**Cognitive Disturbance**	2	6	0	0	**8 (3.48%)**
**Concentration Impairment**	5	6	0	0	**11 (4.78%)**
**Confusion**	2	0	1	0	**3 (1.30%)**
**Constipation**	11	0	0	0	**0 (0%)**
**Cough**	1	0	0	0	**0 (0%)**
**Delirium**	1	0	0	0	**1 (0.43%)**
**Depression**	0	1	0	0	**1 (0.43%)**
**Diarrhoea**	1	2	0	0	**3 (1.30%)**
**Dizziness**	7	4	2	0	**13 (5.65%)**
**Dry Mouth**	11	5	0	0	**16 (6.96%)**
**Dysgeusia**	4	0	1	0	**5 (2.17%)**
**Dyspepsia**	3	2	0	0	**5 (2.17%)**
**Fall**	0	0	0	0	**0 (0%)**
**Fatigue**	2	5	8	0	**15 (6.52%)**
**Fever**	2	0	0	0	**2 (0.87%)**
**Flu-like symptoms**	0	0	0	0	**0 (0%)**
**Generalised Muscle Weakness**	3	3	2	0	**8 (3.48%)**
**Headache**	4	6	2	0	**12 (5.22%)**
**Increased appetite**	0	0	1	0	**1 (0.43%)**
**Insomnia**	4	6	3	0	**13 (6.65%)**
**Lethargy**	4	8	0	0	**12 (5.22%)**
**Mucositis Oral**	0	0	1	0	**1 (0.43%)**
**Muscle Cramps**	0	1	0	0	**1 (0.43%)**
**Nausea**	8	3	0	0	**11 (4.78%)**
**Otitis Externa**	0	0	0	0	**0 (0%)**
**Palpitations**	0	0	0	0	**0 (0%)**
**Pharyngitis**	0	4	0	0	**4 (1.73%)**
**Post COVID19 Syndrome**	0	0	0	0	**0 (0%)**
**Rash**	0	0	0	0	**0 (0%)**
**Somnolence**	0	15	0	0	**15 (6.52%)**
**Spasticity**	0	0	1	0	**1 (0.43%)**
**Stevens-Johnson Syndrome**	0	0	1	0	**1 (0.43%)**
**Tremor**	2	1	0	0	**3 (1.30%)**
**Urinary Tract Infection**	0	2	0	0	**2 (0.87%)**
**Uveitis**	0	1	0	0	**1 (0.43%)**
**Vertigo**	5	2	1	0	**8 (3.48%)**
**Vomiting**	0	0	1	0	**1 (0.43%)**
**Weight Gain**	0	1	1	0	**2 (0.87%)**
**Weight Loss**	1	0	0	0	**1 (0.43%)**
**Total (%)**	**102 (44.35%)**	**97 (42.17)**	**31 (13.48%)**	**0 (0%)**	**230 (100%)**
